# Identifying Political Sentiments on YouTube: A Systematic Comparison Regarding the Accuracy of Recurrent Neural Network and Machine Learning Models

**DOI:** 10.1007/978-3-030-61841-4_8

**Published:** 2020-10-19

**Authors:** Daniel Röchert, German Neubaum, Stefan Stieglitz

**Affiliations:** 8 Leiden Institute of Advanced Computer Science, Leiden University, Leiden, The Netherlands; 9 Leiden Institute of Advanced Computer Science, Leiden University, Leiden, The Netherlands; 10 School of Politics and International Studies, University of Leeds, Leeds, UK; 11 Leiden Institute of Advanced Computer Science, Leiden University, Leiden, The Netherlands; 12 Leiden Institute of Advanced Computer Science, Leiden University, Leiden, The Netherlands; University of Duisburg-Essen, 47057 Duisburg, Germany

**Keywords:** Deep learning, Machine learning, Text classification, Word embeddings, Computational science

## Abstract

Since social media have increasingly become forums to exchange personal opinions, more and more approaches have been suggested to analyze those sentiments automatically. Neural networks and traditional machine learning methods allow individual adaption by training the data, tailoring the algorithm to the particular topic that is discussed. Still, a great number of methodological combinations involving algorithms (e.g., recurrent neural networks (RNN)), techniques (e.g., word2vec), and methods (e.g., Skip-Gram) are possible. This work offers a systematic comparison of sentiment analytical approaches using different word embeddings with RNN architectures and traditional machine learning techniques. Using German comments of controversial political discussions on YouTube, this study uses metrics such as F1-score, precision and recall to compare the quality of performance of different approaches. First results show that deep neural networks outperform multiclass prediction with small datasets in contrast to traditional machine learning models with word embeddings.

## Introduction

On social media platforms such as YouTube, Facebook, or Twitter, a mass of people interact with each other on a daily basis, commenting on media content such as videos and exchanging their viewpoints on different issues. Since politically and civically relevant communication is becoming more and more prevalent on social media, identifying opinion climates and optimizing approaches remains as an important task for research. To identify the most appropriate method that detects sentiments in political discussions is of pivotal relevance when it comes to grasp dysfunctional communication processes online. For instance, knowing how different opinions are related to each other contributes to assess to what extent politically homogeneous/heterogeneous cocoons exist. Besides this, identifying sentiments among social media users could also help to asses the opinion climate toward misinformation and to examine that dynamics such misinformation can induce in certain networks. Cross-user generated content such as comments, likes, dislikes or related videos are exchanges of information on a specific topic, also in multi-language context and contain many additional metadata that can be used to analyze user behavior and their current sentiment of a specific topic. Sentiment analysis (SA) also known as opinion mining as a particular form of natural language processing (NLP) is a common tool to grasp communication patterns on social media and is becoming progressively relevant in the research area of social media analytics [34]. Challenges in the area of NLP refer to understanding and processing human communication by machines, not by fixed rules or dictionaries, but rather by training them to learn these complex natural languages. The utilization of SA has become an important method in various domains: product reviews, movie reviews, election campaigns, stock market prediction and social media behavior analysis. The usage of SAs in social media might be used for the decision-making process of companies in order to trace more accurate product strategies based on the customers’ current opinions. The more precise the outcome of the SA with regard to product or service reviews, the more effectively strategies can be deployed to prevent crises or to adapt customer requirements. Employing a machine learning approach, a recent study estimated that approx. 60–80% of YouTube comments contain opinions [31]. This makes it highly attractive to identify opinion climates with SA techniques investigating not only public opinion on political issues but also brands and products. Given these numbers, the present study relies on user comments gathered on the platform YouTube. We chose YouTube as a communication platform for our study due to the given prevalence of opinion expressions and its worldwide popularity. The present work applied a comparison of deep learning (subset of machine learning) and traditional machine learning techniques for the categorical classification task to predict the user sentiment score in political YouTube comments and their replies with own input weights of pre-trained word embeddings. Artificial neural models have successfully established themselves in other text classification tasks and achieved good results [26]. However, studies systematically comparing different sentiment analytical combinations are still scarce, especially on the social media platform YouTube with German YouTube comments. With this work, we aim to fill this gap and offer one of the first analyses of German comments on YouTube by using different machine learning techniques. Moreover, this study examines which of those techniques provides better results by combining them with recurrent neural networks and machine learning models. To formalize the overall goals of this paper, the following questions are guiding this research:

**RQ1.**: What is the difference in performance of sentiment classification between recurrent neural networks and traditional machine learning methods?
**RQ2.**: Which of the generated word embedding techniques yields the most accurate results for classification and are any differences detectable among these techniques?

First we crawled YouTube data through the YouTube API, pre-processed the comments and replies by removing inconsistent data and transform them into sentences. Afterwards we transformed all of these sentences into one high dimensional vector also called word embedding which amplifies a dense distributed representation for each word in a high dimension space with the frameworks word2vec and fastText by applying two different techniques such as Skip-Gram and Continuous Bag of Words (CBOW). These word embeddings learned the semantic of their surrounding words and will help to train the deep neural network model as well as the machine learning models.

## Theoretical Background

### Related Work

Social media have become important communication channels for public interactions in today’s digital society. Especially, the investigation of political communication on social platforms, which are examined by means of user-generated comments, plays an increasingly important role in different research areas such as hate speech, misinformation, or political homogenization and polarization. These areas are particularly concerned with the dark side of social media and the ever-growing threat to democracy in society [35]. In particular, the topic of hate speech in social media has generated a lot of attention worldwide in the last few years and is still a current problem for service providers. A study analyzed user comments on the refugee crisis in Germany in 2015/2016 on various news portals [15]. In the study, a binary classifier has been trained using logistic regression, which has achieved a F1-score of 0.67. Further, the researchers have been able to show that many hate words refer to political topics. In addition to the mono-linguistic identification of hate speech, there have been attempts to identify hate speech in different languages using deep-learning techniques and compare them to traditional machine-learning methods [23]. Another aspect that relates to the political context of social media is that these platforms are more often portrayed as a threat to democracy, as they allow interactions between like-minded people. A recent study has examined the YouTube discussion network of comments using opinion-based homogeneity to identify the climate of opinion [29]. The results of the study show that YouTube users reply less on political comments that reflects their own position than on comments that reflect a different opinion. A further problem with social media is that it allows any person to spread claims without any fact-checking. Previous research has shown that the use of social media can increase the impact of fake news and that the main purpose of social media is to influence public opinion as well as political events [18]. In order to prevent this spread of misinformation, several studies focus on the dissemination and detection of misinformation. A recent study has investigated the identification of fake news from text and images on Twitter using convolutional neural networks and recurrent neural networks [1]. The recurrent neural network has achieved the best performance, thereby making it possible to identify relevant features that are classified as fake news. These “hot topics” in computational research indicate that there is a need to identify those analytical approaches that yield the best classification of sentiments within the large amount of communication data in social media.

### Recurrent Neural Network

Recurrent Neural Networks (RNN) [30] are used for processing sequential information such as language modeling, machine translation, time series prediction or image captioning. The general idea of RNNs is to create a kind of “memory” by performing the same operations on every input values in a feedback connection. This process allows to remember the network from previous processed information by sharing the same weights (parameter sharing) across several time steps in the hidden state and perform the output which depends on the passed information to next network [7]. Especially in NLP, this feature is quite helpful to process sequence of sentences because they mainly follow the same rules across the sequence. Parameter sharing makes it possible to perform the same task at each time-step with different input sequences of variable length and makes it therefore more powerful and dynamic compared to normal feed forward neural networks. It reduces the total number of parameters, which means the RNN does not have to learn the same rules of sequences again and already knows their weights. The formula for processing of sequences of a vector $x$ at every time step looks as follow:

1 $$\begin{aligned} h_t = fW(h_{t-1}, x_t) \end{aligned}$$

where the activation function $f$ will depend on weights $W$, which accepts the previous hidden state $h_{t-1}$ as well as the input at the current state $x_t$. This output will the updated hidden state called $h_t$.

### Word Embeddings

In 1954, Zellig Harris established the hypothesis that the difference in meaning correlates with the difference in distribution, also known as the distributional hypothesis [10]. This hypothesis is grounded by distributional semantics, which is an active area of research in natural language processing to develop new techniques to capture various semantic phenomena, by computing semantic similarities between words based on their distributional properties in the corpus. One of these techniques is called word embedding and describes the mapping process of words from a vocabulary into a high dimensional vector spaces by keeping semantically related words close together. It uses an embedding matrix $E \in \mathbb {R}^{|V|\times d_w}$ where $d_w$ is the dimensionality of the embedding space and $|V|$ is the size of the vocabulary. In previous research, this technique is an efficient way to improve and simplify many NLP applications such as machine translation [20, 39], spelling correction [13] or SA [4, 17]. In the context of a SA with classification problem, word embeddings are mainly used to include the semantic connections of words in the analysis to develop better and more accurate predictions. A widely used unsupervised word embedding algorithm is called word2vec^1^, which has been developed by Mikolov et al. from Google and contains a two-layer neural network, which uses text data as input and transforms the output as a set of high dimensional vectors [19]. Another unsupervised distribution semantic model is called fastText^2^ which has been developed by Facebook and is essentially an extension of word2vec model. The main difference of both methods is that the fastText algorithm supports the use of n-grams, which improve the syntatics tasks by taking morphological information into account [3]. Both models have implemented the CBOW and the Skip-Gram methods for computing vector representations of words and are based on hierarchical softmax and negative sampling. The Skip-Gram method has been introduced by Mikolov et al. and predicts potential neighboring words based on a target word [19]. Whereas the CBOW technique uses the context of the neighboring words and predicts the target word. Negative sampling is a modification of an approach called Noise Contrastive Estimation (NCE) [8]. The main idea of the sampling-based approach is to reduce the performance of computational by noise contrastive estimation with several negative examples. An experiment has shown that the negative sampling method is the most efficient algorithm independent from the language used [22]. The present work is intended to compare different combinations of techniques (word2vec and fastText) and methods (Skip-Gram and CBOW), generating unique word vectors that represent the projection of YouTube comments in a continuous vector space.

## Research Method

**Fig. 1.**
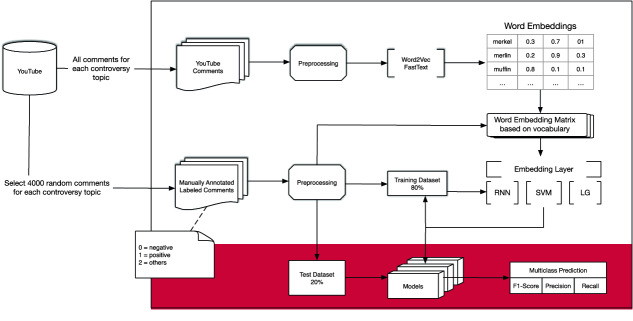
Process of training and evaluation.

This section deals with the sentiment analysis by using deep leaning methods such as RNN to analyze two controversial topics in Germany that were discussed on the social media platform YouTube. The following Fig. 1 demonstrates the process structure of the approach. First, we crawled YouTube data through the YouTube API, preprocessed the comments and replies by removing inconsistent data and transform them into sentences. Afterwards, we transformed all of these sentences into one high dimensional vector also called word embedding which amplifies a dense distributed representation for each word in a high dimension space with the frameworks word2vec and fastText by applying two different techniques such as Skip-Gram and CBOW. These word embeddings learned the semantic of their surrounding words and will help to train the model. The recurrent neural network has been used to initialize these embedding weights to train the network and to create a classifier for further predictions.

### Data Acquisition

The data of this study were gathered on YouTube crawled by the YouTube API and conducted on May 15th 2018. The data comprised comments and replies of two controversial topics in Germany. The first crawl started with the search criterion: “Kopftuchverbot in Deutschland - Headscarf ban in Germany”, for this search we collected 320 unique videos with a maximum number of 48,354 comments and replies. The debate “Wearing religious headscarves” is met with supporters who claim that to fulfil freedom of religion it needs to be allowed while opponents state that headscarves are a symbol for female oppression. For the second query, we used the search terms: “Adoptionsrecht für homosexuelle Paare - Adoption rights for homosexual couples” and contains 15,889 comments and replies with 266 unique videos. In Germany, the debate “Adoption rights for homosexual couples” continues to cause debate. Advocates argue that there is no reason to not allow joint adoption for homosexual partners, whereas opponents argue that every child needs a mother and a father, reflecting their normative ideas of family life. Both topics were selected because they highlight current and controversial issues in society and thus have a lot of potential for discussion in social media. We assume that both controversial topics exhibit a sentiment diversity (i.e. a similar distribution of pros and cons). YouTube labels each video with their own categoryID^3^, in this case we use categoryID of 25, which stands for “Politics and News” in the YouTube API. After filtering the comments and replies for both search criterion’s, we had a data pool of 14,277 comments and replies for the first dataset “Headscarves” and 8,443 comments and replies for the second dataset “Adoption rights”.

### Annotator Agreement

We selected two well trained independent annotators who received the same dataset with 4,000 randomly selected comments and replies for each topic. The term “well trained” refers to annotators who have received personal instruction on the topics presented. Furthermore, the explanation of the coding scheme included example sentences and their coding was outlined and clarified. Through the personal instruction, questions and problems could be clarified to eliminate inconsistencies. The data were labelled with one of three classes which were mutually exclusive: *negative*, *positive* and *others* based on an existing coding scheme [29]. While there was 86.6% percent agreement for the topic of headscarf ban, 82.5% of percent agreement was reached for the topic of adoption right for homosexual couples. In order to ensure better results for all machine learning models, we have decided to use only those records that have an equal match between both annotators for later analysis.

### Data Preparation

Besides the labeling process, another complex task is the cleaning of unstructured data. In terms of data preparation, cleaning unstructured data guarantees that algorithms can classify better and compute more accurate results with the pre-prepared data [34]. Therefore, the main workflow of cleaning the text by regular expression includes: removing hyperlinks and usernames; removing special symbols and numerical values; converting words into lowercase and assigning smilies into three different word categories such as “emotionhappy”, “emotionsad”, “emotionlaugh.”

After cleaning the text, it is necessary to split it into sentences or paragraphs, which is required for the word embedding models word2vec and fastText in Python. For the supervised learning problem, it is required to separate the entire dataset into the training and test datasets. Each dataset is split into training set (80%) and test set (20%). This separation has to be randomized to guarantee that there is no noise in the dataset. We used 5-fold cross-validation on the training dataset to evaluate the performance of all models with a fixed combination of manual-based hyperparameters.

### Unsupervised Learning

In our approach, we used a Python implementation of the word2vec and fastText from Gensim, which is used for NLP task like topic modeling, document indexing and similarity retrieval [27]. We decided to generate our own word embeddings because recent studies have shown that the creation of domain-specific word embeddings such as (crisis, patent) in particular can enhance the performance of the classification, compared to the pre-trained embeddings of Wikipedia or Google News, which are more suitable for more general classification tasks [16, 28]. We created for each method 300 high dimensional word embeddings on basis of the comments and replies of the whole corpus where the words represent as unigrams. As mentioned earlier, both models word2vec and fastText apply Skip-Gram and CBOW techniques and use the same parameter settings to make them comparable afterwards in the evaluation of the sentiment model. The applied parameters with a brief description of their functionalities are presented in the following:

*1.*: *size: represents the dimension of the feature vectors.*
*2.*: *min_count: represents the minimum frequency per token to filter rare words.*
*3.*: *alpha: represents the learning rate of the network.*
*4.*: *iter: represents the epoch over the corpus to update the weights.*
*5.*: *sample: represents the threshold for configuring which higher-frequency words are randomly downsampled.*
*6.*: *negative: represents the amount of how many “noise words” should be included during training.*

The final parameters that have been used for all word embeddings are *size* with a value of 300, *min_count* with a value of 5, *alpha* with a value of 0.01, *iter* with a value of 15, *sample* with a value of 0.05, and *negative* with a value of 15. Since our vocabulary has a size of 11,435 and the dataset contains 126,362 clean sentences with 1,939,663 tokens, we have deliberately opted for a larger dimension (300). For the further process, we chose negative sampling as baseline in this work, which can improve the computation of word embeddings for frequent words and also decrease the performance of training speed of the neural network [21]. Table 1 demonstrates the representation of the embedding matrix to find the top four most similar entities for the word “kopftuch” (headscarf) and “homosexuell” (homosexual). Looking at the results of the two methods, it is noticeable that the most similar words of word2vec are rather different, but nevertheless relevant to the context. On the other hand, the entities of fastText consist of many variations that are very close to the actual word. It is therefore relevant to examine to what extent which of the two methods delivers the better results in the prediction.

**Table 1.** Word similarities of the words “kopftuch” and “homosexuell”.

Target word: kopftuch	**word2vec**	
	**CBOW**	**SkipGram**
	hijab (0.68)	tragen (0.66)
	koptuch (0.66)	hijab (0.60)
	kopftücher (0.60)	koptuch (0.59)
	tuch (0.57)	minirock (0.59)
	**FastText**	
	kopftuchs (0.97)	kopftuchzwang (0.82)
	kopftuchgebot (0.97)	kopftuchfrau (0.82)
	kopftuchzwang (0.96)	kopftuchs (0.81)
	Koftuch (0.94)	kopftuchgebot (0.8)
Target word: homosexuell	**word2vec**	
	schwul (0.80)	schwul (0.62)
	heterosexuell (0.79)	lesbisch (0.60)
	bisexuell (0.73)	heterosexuell (0.60)
	lesbisch (0.72)	bisexuell (0.59)
	**FastText**	
	homosexuel (0.97)	homosexuel (0.93)
	homosexuele (0.97)	homosexuele (0.93)
	homosexuelle (0.95)	homosexuellen (0.82)
	homosexuelles (0.95)	homosexuelle (0.82)

### Supervised Learning

For the supervised learning task, we implemented our model based on Keras with a TensorFlow backend [5]. Keras is a Python library for developing deep neural networks. The baseline models have been implemented as well in Python, but with the sci-kit library for machine learning in Python [24]. The implementation and configuration of all recurrent neural networks share all the same parameters to make them comparable with the different combination of word embeddings. We used a many-to-one model for our architecture, where the input of the network is characterized by sentences with variably sized and multiple words. The first layer of our sequential model is the embedding layer initialized by a dimension 300 and an input length of 100. After this layer, we set a recurrent layer with 64 hidden units, an internal dropout rate of 0.1 and a recurrent dropout of 0.1. The main reason for the regularization of a neural network is the increase in performance and its applicability to unseen data beyond the training data and to avoid overfitting. Especially for small datasets, neural networks are more inclined to overfit than on large datasets because they are used to learn from large data. For regularization of our network, we decided to employ to usual methods: applying dropout to the networks [11], using $L_2$ weight regularization as well as class weights. The general idea of dropout is to avoid co-adaptations by applying random dropout units during the training of the neural network [33]. Using dropout can greatly reduce overfitting in RNNs [37]. Besides the dropout regularization, we used $L_2$ weight decay to reduce the complexity of the softmax function. Readjustment of the class weights have been applied to re-balance the classes and make them more reasonable and equally considered during the training. Classes that appear in the dataset often achieve low weights, whereas infrequent classes receive higher weights to re-balance the training. As an optimization function to train our network, we choose the extension to stochastic gradient descent called Adam [14] with the categorical cross entropy loss function, suited to multi-class classification problems. The output layer are characterized by three neurons with a softmax activation function for predicting the probability distribution for each class. Further, we trained the model with a mini-batch size of 10 and set the number of epochs of 100. Because our dataset is small for training and testing we chose a small training batch size, as well as small hidden units of the neural networks to increase the accuracy of the prediction.

### Baseline Models

We have also implemented traditional machine learning models, so that we can identify how neural networks perform in comparison to methods which can perhaps better handle smaller datasets. In a study where Chinese short texts with public financial documents were classified, it was shown that the support vector machines as well as logistic regressions achieved the best results of the performance of the prediction [36]. More precisely, by comparing machine learning models, it was shown that logistic regression in the area of product reviews [25] or BBC news [32] achieved better results than other classical machine learning models such as k-nearest-neighbors or random forest. Apart from logistic regression, there exist also several studies showing that the Support Vector machine was successfully applied for text classification and reached the best performance in multi-class prediction [16, 38]. Based on the positive results of the previously stated studies, we have decided to use SVM and logistics regression as baseline machine learning techniques. In general, machine learning models such as support vector machines or logistic regression cannot directly handle word embeddings, which are represented in a high-dimensional space, therefore we have to prepare our 300 dimensional word embeddings into one dimensional by using the average value of each word vectors. This allows us to represent each word by an average value and have been successfully implemented on other studies [2]. The following machine learning techniques have been performed:

- Support Vector Machines (SVM): are based on the margin maximization principle and used for non-linear and linear regression and classification tasks. The SVM uses a penalty parameter $C$ of the value of 100, which is characterized as the error term, the smaller the value, the stronger is the regulation of the model. As well, we applied a $linear$ kernel and balanced class weights to the model.
- Logistic Regression (LG): as well as SVM, logistic regression is a supervised learning algorithm to estimates the probability of a categorical dependent variables by computing the sigmoid function. Like for the recurrent neural networks, we avoided overfitting by applying $L_2$ weights regularization with the $saga$ solver. Also we used balanced class weights to adjust the imbalanced distribution of classes.

## Results

Since the models have been trained successfully, the information of the models can be extracted and used for analysis. The results for the prediction on the test datasets are shown in Table 2. We used precision, recall and F1-score to measure the performance of three different classes. For multi-class tasks which are imbalanced, it is recommended to apply weighted F1-score, which computes the average for each class. The results for the performance F1-score reveal two main features. First, the results show that in general all recurrent neural networks outperform the machine learning models. The best performance was achieved with RNNs obtained by combining word2vec with CBOW for both datasets. Second, focusing on the different word embedding methods like Skip-Gram and CBOW, the results indicate that CBOW performs better than Skip-Gram, especially for RNNs, but this does not apply to the remaining results. Looking at the results for the individual word embeddings techniques “word2vec” and “fastText”, no particular difference is noticeable because the F1-values are generally very similar to each other.

**Table 2.** Evaluation result of deep learning and traditional machine learning methods on test dataset.

			Adoption rights			Headscarves		
Models	Technique	Method	F1-score	Precision	Recall	F1-score	Precision	Recall
**RNN**	**word2vec**	Skip-Gram	0.715	0.726	0.706	0.789	0.794	0.784
		**CBOW**	**0.746**	**0.748**	**0.744**	**0.823**	**0.815**	**0.835**
	fastText	Skip-Gram	0.724	0.739	0.738	0.754	0.806	0.724
		CBOW	0.741	0.731	0.760	0.755	0.793	0.731
SVM	word2vec	Skip-Gram	0.565	0.717	0.509	0.543	0.798	0.470
		CBOW	0.568	0.721	0.512	0.543	0.798	0.470
	fastText	Skip-Gram	0.567	0.719	0.512	0.544	0.801	0.471
		CBOW	0.560	0.723	0.503	0.552	0.798	0.480
LG	word2vec	Skip-Gram	0.597	0.704	0.550	0.647	0.783	0.585
		CBOW	0.592	0.703	0.544	0.642	0.783	0.577
	fastText	Skip-Gram	0.600	0.710	0.553	0.649	0.783	0.587
		CBOW	0.581	0.699	0.532	0.629	0.773	0.562

## Discussion

This study offered a systematic comparison of combinations consisting of different sentiment analytical approaches such as deep neural networks and machine learning models. With regard to RQ1, we can conclude that our approach has demonstrated that the artificial neural network models outperform usual machine learning models by embedding high dimensional vectors. In order to have a fair comparison, hyperparameters were kept constant in this study. The fact that deep neural networks generally reach higher F1-scores may be explained by different factors: First, the weaker results of machine learning methods can be explained by the fact that they cannot capture the high-dimensional word vectors during training, but only receive averaged word vectors for all words in the corpus. As a result, important information is no longer provided during the computation and performance deteriorates. Second, deep neural networks might reach a higher level of accuracy when hyperparameters are determined by grid search or random search in accordance with the dataset at hand. Third, it must be noted that the dataset is imbalanced, methods to weight the classes are beneficial, but more effective would be actual datasets with equally distributed classes. Given that the category with the most comments was the *others* class, all methods might benefit more from a dataset that has a larger portion of positive versus negative comments whose context are easier to identify (than from *others* comments). When considering the normalized confusion matrix, the class most frequently predicted in neural networks is “others”, which therefore has a positive effect on the F1-score, since this class is most frequently represented in the dataset. Regarding RQ2, it can be concluded that word embeddings have significantly improved the performance of RNN compared to the traditional ML models. Due to the different models, however, it is not possible to determine exactly which method and technique is the best because the different combinations of word embeddings have computed relatively similar outcomes.

## Further Research

To conclude, the present study revealed that with small datasets of user comments on YouTube, deep neural networks outperform machine learning models. For future work, it would be interesting to improve some features to achieve more precise results in the prediction. The first improvement in analysis might consist of applying advanced models and techniques to compute even more accurate predictions. Simple RNNs are often used for processing long-term sequences like documents, however studies have shown that RNNs are mainly suitable for short term dependencies because of the vanishing gradient or exploding gradient problem [12], which makes them inaccurate for tasks that require long-term sequences. This problem appears when training deep neural networks to learn dependencies by backpropagation through time over long time steps, which can reach extremely high or exponentially small values of gradients. To avoid this kind of problem, it is commendable to apply other recurrent neural network architectures such as long-short term memory. For further research, it would be advisable to implement and compare the improved RNNs like long-short term memory networks as well. Furthermore, it would be a reasonable idea to utilize further machine learning algorithms such as naive bayes or random forest, which are not based on word embeddings but on term frequency times inverse document frequency vectors to extend the systematic comparison and test which combined approaches offer more accurate results. It does not require word embedding but is also used for SA [6, 9]. While word embedding links the semantics of sentences, term frequency times inverse document frequency computes the importance of a term inside a comment by their frequency of the entire dataset. In addition, a further aspect that should be considered when using word embeddings in future research is the comparison with already existing pre-trained models such as Wikipedia or Google News to be able to make semantic comparisons between these and own domain-specific models and to take into account which models are better suited for classification. Another necessary step is to perform SA with other languages in order to achieve greater diversity and compare them against each other. In addition to political and controversial topics, it would also be appropriate to collect data from product reviews or unboxing videos and evaluate the comments with the aid of SA to gain experience in this field as well.

## Footnotes

1 https://code.google.com/archive/p/word2vec/.

2 https://fasttext.cc.

3 https://developers.google.com/youtube/v3/docs/videoCategories.
